# Structural basis of DNA synthesis opposite 8-oxoguanine by human PrimPol primase-polymerase

**DOI:** 10.1038/s41467-021-24317-z

**Published:** 2021-06-29

**Authors:** Olga Rechkoblit, Robert E. Johnson, Yogesh K. Gupta, Louise Prakash, Satya Prakash, Aneel K. Aggarwal

**Affiliations:** 1 Department of Pharmacological Sciences, Icahn School of Medicine at Mount Sinai, New York, NY USA; 2 Department of Biochemistry and Molecular Biology, University of Texas Medical Branch, Galveston, TX USA; 3 Present Address: Greehey Children’s Cancer Research Institute & Department of Biochemistry and Structural Biology, University of Texas Health at San Antonio, San Antonio, TX USA

**Keywords:** Structural biology, Translesion synthesis, X-ray crystallography

## Abstract

PrimPol is a human DNA polymerase-primase that localizes to mitochondria and nucleus and bypasses the major oxidative lesion 7,8-dihydro-8-oxoguanine (oxoG) via translesion synthesis, in mostly error-free manner. We present structures of PrimPol insertion complexes with a DNA template-primer and correct dCTP or erroneous dATP opposite the lesion, as well as extension complexes with C or A as a 3′−terminal primer base. We show that during the insertion of C and extension from it, the active site is unperturbed, reflecting the readiness of PrimPol to accommodate oxoG*(anti)*. The misinsertion of A opposite oxoG*(syn)* also does not alter the active site, and is likely less favorable due to lower thermodynamic stability of the oxoG*(syn)*•A base*-*pair. During the extension step, oxoG*(syn)* induces an opening of its base-pair with A or misalignment of the 3′-A primer terminus. Together, the structures show how PrimPol accurately synthesizes DNA opposite oxidatively damaged DNA in human cells.

## Abstract (web summary)

The human DNA primase and DNA polymerase PrimPol replicates through the major oxidative DNA damage lesion 7,8-dihydro-8-oxoguanine (oxoG) via translesion synthesis in a mostly error-free manner thus suppressing oxoG-induced mutagenesis in mitochondria and the nucleus. Here, the authors present crystal structures of PrimPol in complex with an oxoG lesion in different contexts that provide mechanistic insights into how PrimPol performs predominantly accurate synthesis on oxidative-damaged DNAs in human cells.

## Introduction

7,8-dihydro-8-oxoguanine (oxoG) is the major oxidative DNA damage lesion in human cells. The oxidative damage is induced by reactive oxygen species (ROS) arising due to normal metabolic processes as well as environmental exposures such as ionizing radiation and the UVA band of sunlight^1–4^. It is estimated that each cell in a human body develops ~2,800 oxoG lesions per day^5^.

OxoG lesion is highly mutagenic because nuclear and mitochondrial (mt) replicative DNA polymerases effectively pair an oxoG template residue not only with the correct cytosine base but also mispair it with an adenine base and preferentially extend from the misinserted primer termini^6–13^. This induces a G to T transversion mutation upon the next round of replication. Since mitochondria produce substantial amounts of ROS in the cell^14^, oxidative damage to the mtDNA is deemed to be especially detrimental^15^. Unexpectedly, recent sequencing studies reveal that the mutations observed in mtDNA are transitions rather than transversions and, thus, are unlikely to be associated with the oxoG-driven mutagenesis^16–20^.

PrimPol is the only human DNA polymerase that is known to localize to mitochondria and is able to efficiently and mostly accurately bypass an oxoG via translesion synthesis (TLS)^21–25^. Consequently, it is expected that PrimPol aids the limited DNA repair processes operating in mitochondria^26,27^ to protect mtDNA from the ROS-induced mutations.

PrimPol is important for both nuclear and mtDNA replication and belongs to the archaeo-eukaryotic primase-polymerase family that differs from other DNA polymerases by possessing a primase activity^21,24,28–32^. Thus, in addition to the catalytic core, PrimPol contains a zinc finger module that is required for the primase function^28,31,33^. The dual TLS polymerase and primase activities of PrimPol allow it to bypass DNA lesions via TLS as well as to ‘skip’ DNA lesions and initiate DNA synthesis de novo downstream of the lesion site. Indeed, PrimPol-primed DNA synthesis re-initiation after multiple cisplatin therapy doses in BRCA1-deficient cancer cells has emerged as a major chemoresistance pathway^34^. The crystal structure of the catalytic core of PrimPol demonstrates that the active site of PrimPol is relatively constrained and is able to accommodate smaller lesions, such as oxoG, and process them via TLS, but is not likely to do so with bulkier DNA adducts, such as UV-induced cis-syn T-T dimer, (6–4) T-T photoproduct or cisplatin-DNA adducts^35^.

DNA polymerases can manipulate the *anti-syn* conformational equilibrium of the glycosidic torsion angle of the oxoG residue and thus governing its pairing either with a C base via its Watson−Crick edge (when in the *anti* conformation) or with an A via its Hoogsteen edge (when in the *syn* conformation). Predominantly error-free oxoG bypass polymerases such as human X-family Polλ^36^ and TLS Y-family archaea Dpo4^37^ are able to favorably accommodate a local template distortion needed to resolve an otherwise close contact between the ‘extra’ O8 atom and the oxygen atoms of the sugar-phosphate backbone of the oxoG residue. Alternatively, human or yeast TLS Y-family Polη^38^ is preconfigured in the oxoG*(anti)*-permissive conformation even on the unmodified template^39,40^. In contrast, within the constrained active sites of high-fidelity A-family DNA Pol I fragment^10^ and Pol T7^9^, the oxoG(*syn)*•A mismatch mimics a cognate base pair and allows for misinsertion and further extension, while the conformational adjustments associated with the correct oxoG(*anti)*•C pair disrupt the active site and stall the polymerase. Mitochondrial replicative Polγ also belongs to the A-family^41^, and is strongly inhibited by the oxoG lesion and predominantly extends from the oxoG•A base pair^11,42^.

PrimPol also stands out among DNA polymerases due to its unusual architecture and minimal contacts with template-primer DNA duplex^32,35^. Instead of the classical fingers-palm-thumb domains observed in all other DNA polymerases, PrimPol catalytic core has two modules: an N-terminal ModN and a C-terminal ModC^35^. ModN makes limited interactions primarily with the template DNA strand and the templating base, whereas ModC contains the active site and interacts with the nascent base pair. Due to the absence of the ‘thumb’ domain, there are almost no contacts to the DNA primer strand. There is also no polymerase-associated domain (PAD, also termed ‘little finger domain’) that provides an extensive interface with the major groove of the template-primer duplex in all Y-family TLS polymerases^32^. Altogether, it is unclear how PrimPol governs up to six-fold preferable insertion of a C over A opposite the oxoG and a two-fold preferable extension from the oxoG•C over the oxoG•A base pair^22^.

Here we present four crystal structures of PrimPol that capture the insertion of a C and an A opposite the oxoG lesion, as well as the subsequent extension from the inserted bases. We show that during the insertion of C and extension from it, the overall conformation of the active site remains the same as in the undamaged complex; thus, demonstrating the readiness of PrimPol to accommodate the oxoG*(anti)* during both steps of the lesion bypass without considerable adjustment to the template DNA backbone. Our results also suggest that the misinsertion of an A opposite the oxoG is likely impaired by the lower thermodynamic stability of the oxoG*(syn)*•A*(anti)* base-pair rather than structural alterations. However, during the next extension step, the 3′-A primer terminus opposite the oxoG*(syn)* lesion can assume either productive or non-catalytic alignment, and thereby reducing the reaction rate. Together, the structures define the basis of PrimPol’s ability to perform predominantly accurate synthesis on oxidative-damaged DNAs in human cells.

## Results

### Structure determination

To produce the insertion ternary PrimPol complexes with the correct C incoming nucleotide triphosphate opposite the oxoG lesion, we crystallized the catalytic core of human PrimPol (residues 1−354) with a 17-nucleotide (nt) DNA template (5′-CA(oxoG)CGCTACCACACCCC-3′) and a 2′-deoxy-3′-terminated 12 nt DNA primer (5′-GGTGTGGTAGCG-3′) in the presence of dCTP. The last two 5′-CC-3′ nucleotides of the template strand from its 3′-end are intentionally left unpaired with the primer strand to promote crystal-packing interactions. The conditions for crystal growth include only Ca^2+^ ions, which do not support catalysis by PrimPol^22^. To produce crystals in the presence of dATP, we employ the aforementioned 17 nt template and 2′-deoxy- or 2′,3′-dideoxy-3′-terminated 13 nt DNA primer, namely 5′-GGGTGTGGTAGCG-3′ or 5′-GGGTGTGGTAGCG_dd_-3′. The 13 nt primer produces a single C nt 3′ template base overhang. To capture the extension step we used a 17 nt DNA template (5′-CAT(oxoG)CCTACCACACCCC–3′), where the oxoG lesion is moved one base downstream compared to its location in the insertion complex, and 13 nt DNA primers with either a C or an A 2′-deoxy-3′-terminal bases (5′-GGGTGTGGTAGGX-3′, where X is C or A), and the next correct incoming base dATP. The structures were solved by molecular replacement using the unmodified ternary complex with T-dATP nascent base pair as a search model (PDB ID: 5L2X)^35^ and are refined to 2.60−2.07 Å resolution range. All structures have two ternary complexes (molecules A and B) in the crystallographic asymmetric unit (AU) that are very similar to each other, except for the N-helix (residues 1−17), which is disordered in the complex B molecules. The crystal data, data collection statistics, and refinement statistics for all complexes are summarized in Table 1.

**Table 1:** X-ray data collection and refinement statistics.

Complex name	oxoG•dCTP	oxoG•dATP	oxoG•dATP	oxoG•C	oxoG•A
Complex type	insertion	insertion	insertion	extension	extension
Primer strand 3′	2′-deoxy	2′,3′-dideoxy	2′-deoxy	2′-deoxy	2′-deoxy
PDB ID	7JK1	7JKL	7JKP	7JL8	7JLG
*Data collection*					
Space group	P1	P1	P1	P1	P1
Cell dimensions:					
* a, b, c* (Å)	52.6 65.7 75.2	52.5 65.6 74.3	52.6 65.7 75.2	50.7 65.3 72.0	51.0 65.2 73.1
* α*, *β*, *γ* (°)	69.7, 82.8, 88.5	69.7, 82.8, 88.5	69.7, 82.8, 88.5	68.5, 85.7, 86.9	71.4, 84.3, 88.6
Resolution range (Å)^a^	70.0−2.62 (2.74−2.62)	69.2−2.37 (2.46−2.37)	69.2−2.58 (2.69−2.58)	28.2−2.10 (2.16−2.10)	27.0−2.07 (2.13−2.07)
*R*_merge_ (%)	11.2 (122)	9.2 (91.7)	8.8 (95.5)	5.5 (54.3)	6.1 (108)
*I* /σ*I*	6.1 (0.9)	7.0 (0.8)	7.6 (0.7)	11.6 (1.7)	8.8 (0.8)
Completeness (%)	98.0 (93.9)	91.2 (64.0)	94.0 (82.1)	97.1 (93.6)	97.0 (95.5)
Redundancy	2.1 (2.1)	3.5 (2.9)	3.4 (2.7)	3.4 (3.3)	3.4 (3.3)
CC_1/2_ (%)	98.8 (36.3)	99.3 (44.9)	99.5 (52.3)	99.8 (78.0)	99.8 (33.6)
*Refinement*					
Resolution range (Å)	39.5−2.62 (2.71−2.62)	69.2−2.38 (2.45−2.38)	69.2−2.58 (2.68−2.58)	28.2−2.10 (2.15−2.10)	27.0−2.07 (2.12−2.07)
No. reflections	27,566	32,954	26,648	48,566	52,522
*R*_work_/*R*_free_	23.9/ 28.6 (36.9/ 38.5)	22.0/ 27.7 (38.6/39.0)	24.8/ 30.7 (48.1/ 50.0)	18.7/ 21.3 (29.2/ 32.2)	20.1/ 21.9 (37.6/ 40.2)
No. of non-hydrogen atoms	5,440	5,483	5,291	5,171	5,351
Protein	4,122	4,159	4,053	4,085	4,116
DNA	1,143	1,143	1,145	807	1,021
Ligand (dNTP)	56	60	60	60	60
Ligand (other)	25	12	6	55	19
Ion (Ca^2+^)	2	3	3	3	4
Water	92	106	24	161	131
*B*-factors					
Protein	64.4	66.0	76.6	52.2	61.1
DNA	115.3	111.1	145.9	114.5	124.0
Ligand (dNTP)	53.9	57.0	70.5	43.3	56.2
Ligand (other)	56.9	62.7	76.9	70.6	74.3
Ion (Ca^2+^)	51.7	74.4	74.6	52.1	73.4
Water	54.2	60.7	63.6	48.9	54.7
*R.m.s. deviations*					
Bond length (Å)	0.011	0.005	0.011	0.008	0.003
Bond angles (°)	0.681	0.497	0.495	1.011	0.647

^a^Values in parentheses are for highest-resolution shell.

### Correct C insertion opposite oxoG

**Fig. 1:**
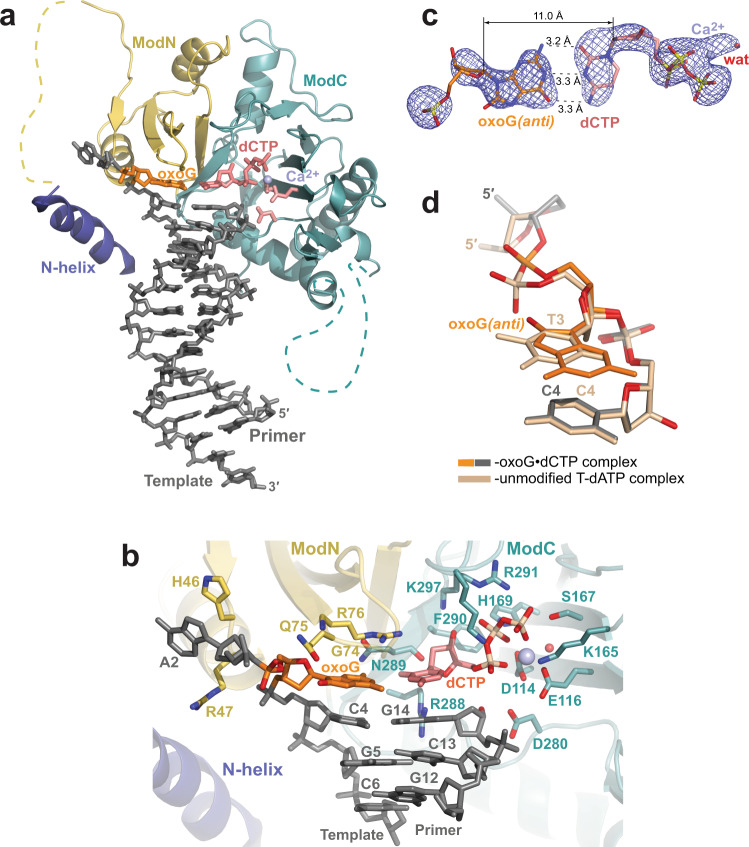
Structure of human PrimPol inserting correct dCTP opposite the oxoG template base. **a** The overall structure of the ternary PrimPol oxoG•dCTP complex (molecule A complex in the asymmetric unit (AU) with the ordered N-helix). The N-helix and modules ModN and ModC are shown in cartoon representation in dark blue, yellow, and cyan, respectively. The DNA is shown as gray sticks, and the Ca^2+^ ion (‘metal B’) is shown as a light blue sphere. The oxoG residue (orange) is forming a nascent base pair with the incoming dCTP (red). The unstructured regions in the ModN and ModC modules are shown as dashed yellow and cyan lines, respectively. The side chains of key catalytic active-site residues Asp114, Glu116, and Asp280 are shown as red sticks. **b** A close-up view of the oxoG template base and the dCTP in the PrimPol active site. The key catalytic residues (Asp114, Glu116, and Asp280), the residues contacting the incoming dCTP (Lys165, Ser167, His169, Arg288, Asn289, Phe290, Arg291, and Lys297), the template oxoG base (Gly74, Gln75, and Arg76), and the rest of the template strand (Lys10, His46, and Arg47) are shown in sticks and with oxygen atoms in bright red and nitrogen atoms in blue. **c** A simulated annealing Fo−Fc omit map (contoured at 2.5σ-level at 2.60 Å resolution and colored in blue) showing the clear electron density for the entire oxoG residue in the *anti* conformation, its partner incoming dCTP, and the Ca^2+^ ion. Hydrogen bonds are indicated by black dashes. **d** Comparison of the phosphate backbone conformations of C2-oxoG3*(anti)*-C4 (gray and orange) and unmodified C2-T3-C4 (beige) template strand segments within the PrimPol active site of their respective ternary complexes. The complexes are superimposed by ModN and ModC of PrimPol protein. The oxygen atoms of the sugar-phosphate backbone and the O8 of the oxoG residue are colored in red. The phosphate group of the oxoG3*(anti)* residue is shifted by ~1.5 Å compared to the position occupied by the phosphate group of T3 residue.

The overall structure of the PrimPol complex depicting insertion of the correct C opposite the template oxoG residue (oxoG•dCTP) is refined to 2.60 Å resolution and is similar to the structure of the unmodified complex (T-dATP) (root mean square deviation (rmsd) of ~0.35 Å over 249 Cαs of molecule A complexes) (Fig. 1a and Supplementary Fig. 1). The PrimPol catalytic core clasps the template-primer by two *α*/*β* modules, ModN and ModC (Fig. 1b). ModN (residues 35−105) interacts primarily with the template DNA strand and the template oxoG base, whereas ModC (residues 108−200 and 261−348) carries the catalytic residues Asp114, Asp116, and Glu280 and interacts with the incoming dCTP nucleotide: functions normally associated with the finger and palm domains of DNA polymerases, respectively. In the absence of the ‘thumb’ domain, there are almost no contacts to the DNA primer strand.

Only one ‘metal B’ Ca^2+^ ion, chelated by the oxygen atoms of the triphosphate tail of dCTP, is observed in the oxoG•dCTP complex despite the intact 3′-OH primer terminus (Fig. 1a, b). A single ‘B’ ion is also present in the unmodified complex with a dideoxy-terminated primer terminus (Supplementary Fig. 1a).

Somewhat different from the unmodified complex, the N-terminal helix (N-helix; residues 1−17) is slightly shifted (by ~2.5−3 Å) and rotated (by ~3°) away from the major groove of the DNA duplex (Supplementary Fig. 1a), allowing for the formation of a salt bridge between the side chains of Arg3 of the N-helix and Glu105, the last C-terminal residue of ModN (Supplementary Fig. 1b). The side chain of Lys10, the only N-helix residue interacting with an oxygen atom of the sugar-phosphate DNA backbone (template residue G5) in the unmodified complex is disordered in the electron density map in the oxoG•dCTP complex. Thus, in addition to the flexibility of the long linker (residues 18 to 34, disordered electron map density) connecting ModN to N-helix, the alignment of the N-helix is itself variable in PrimPol. The N-helix bears some resemblance to the PAD (or the little finger domain) in Y-family polymerases^32^ by way of its location and placement in the DNA major groove, but it lacks the multiple contacts that the PAD forms with the backbone of the DNA. Further, we observe ordered electron density for the PrimPol-unbound end of the DNA duplex, which is engaged in end-to-end DNA−DNA interactions via two stacking two-tier triplexes that promote crystal formation (Supplementary Note 1 and Supplementary Fig. 2).

The template oxoG base assumes the *anti* conformation about the glycosidic bond and forms Watson−Crick (WC) interactions with the incoming dCTP (Figs. 1b, c). The C1′−C1′ distance in the nascent oxoG*(anti)*-dCTP base pair is 11 Å (Fig. 1c), which is slightly longer than the C1′−C1′ distance in the T-dATP pair of the unmodified PrimPol complex (10.4 Å) and in a standard WC pair (10.5 Å). The phosphate group of the oxoG*(anti)* shifts by ~1.5 Å to avoid a close contact between the O8 and the O5′ atoms of the oxoG residue (Fig. 1d), and thereby resolving the intrinsic steric clash of the oxoG residue in the *anti* conformation. That is, this oxygen-to-oxygen atom distance would be too close (at ~2.6 Å) if the placement of the phosphate group was preserved as it is for the template base in the unmodified PrimPol complex (Fig. 1d). Thus, the O8 and the O5′ of the oxoG are now located ~3.5 Å from each other. Since the phosphate group of the template residue in either complex does not make any interactions with PrimPol protein residues, the shift of the phosphate group has little implication for catalytic alignment. This is reflected in the relatively modest reduction (~26%) in the efficiency of incorporation of dCTP opposite the oxoG compare to the unmodified G template base^22^.

### Mutagenic A insertion opposite oxoG

**Fig. 2:**
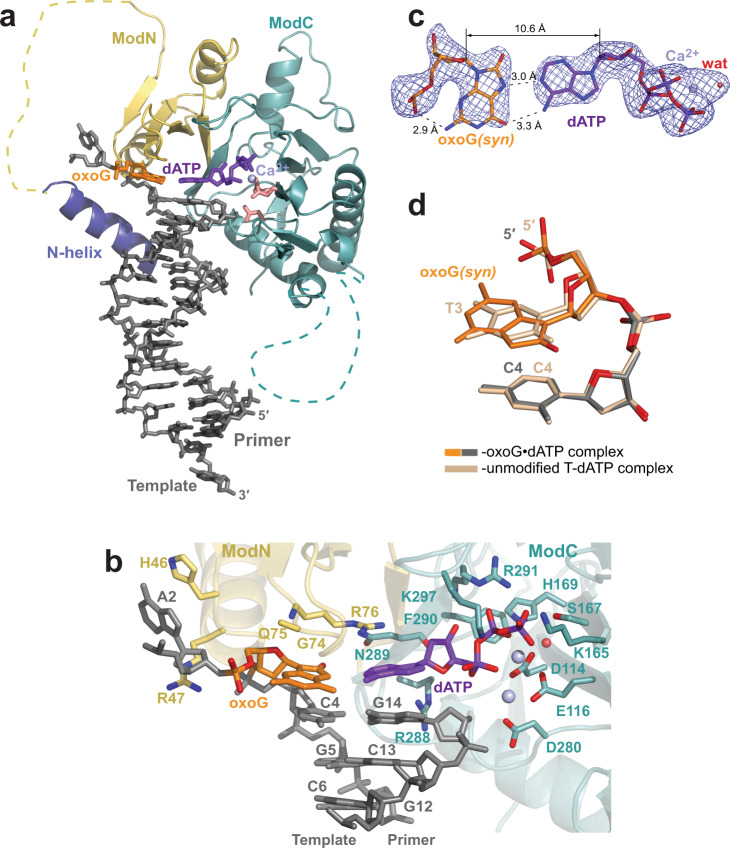
Structure of human PrimPol inserting incorrect dATP opposite the oxoG template base. **a** The overall structure of the ternary PrimPol oxoG•dATP complex with the 2′,3′-dideoxy 3′-terminated primer (molecule A of the AU). The N-helix and modules ModN and ModC are shown in cartoon representation in dark blue, yellow, and cyan, respectively. The DNA is shown as gray sticks, the Ca^2+^ ion (‘metal B’) is shown as a light blue sphere, and the water molecule coordinated by the Ca^2+^ ion is shown as a small red sphere. The oxoG residue (orange) is forming a nascent base pair with the incoming dATP (purple). The unstructured regions in the ModN and ModC modules are shown as dashed yellow and cyan lines, respectively. The side chains of key catalytic active-site residues Asp114, Glu116, and Asp280 are shown as red sticks. **b** A close-up view of the oxoG template base and the dATP in the PrimPol active site of the 2′-deoxy-3′-terminated primer (intact 3′-OH group)-containing complex (molecule B of the AU). There are two Ca^2+^ ions (metals ‘A’ and ‘B’) in this complex. The key catalytic residues (Asp114, Glu116, and Asp280), the residues contacting the incoming dCTP (Lys165, Ser167, His169, Arg288, Asn289, Phe290, Arg291, and Lys297), the template oxoG base (Gly74, Gln75, and Arg76), and the rest of the template strand (Lys10, His46, and Arg47) are shown in sticks and with oxygen atoms in bright red and nitrogen atoms in blue. The side chains of Gly74 and Gln75 are disordered in this complex and, thus, not shown. **c** A simulated annealing Fo−Fc omit map (contoured at 2.5σ-level at 2.40 Å resolution and colored in blue) showing the clear electron density for the entire oxoG residue in the *syn* conformation, its partner incoming dATP and the Ca^2+^ ‘metal B’ ion observed in the complex with the 2′,3′-dideoxy 3′-terminated primer. Hydrogen bonds are indicated by black dashes. Such oxoG*(syn)*•dATP base pair arrangement is observed in all mentioned PrimPol oxoG•dATP complexes. **d** Similar phosphate backbone conformations of C2-oxoG3*(syn)*-C4 (gray and orange) and unmodified C2-T3-C4 (beige) template strand segments within the PrimPol active site of their respective ternary complexes. The complexes are superimposed by ModN and ModC of PrimPol protein.

We obtained two PrimPol complexes depicting the insertion of dATP opposite the oxoG template residue. The first complex has a 2′,3′-dideoxy-3′-terminated primer and is solved to higher 2.4 Å resolution, and the second complex contains a primer with an intact 3′-OH end and is solved to lower 2.6 Å resolution. The overall structures of both complexes are very similar to the unmodified structure (Fig. 2a and Supplementary Fig. 3a) and to each other (Supplementary Fig. 3b), including the placement of the N-helix. The end-to-end DNA−DNA duplex packing interactions, producing a three-tier triple helix, are well-resolved (Supplementary Note 2 and Supplementary Fig. 4).

There are two Ca^2+^ ions in molecule B of the oxoG•dATP 3′-OH-primer-containing complex (Fig. 2b), corresponding to ‘metal A’ and ‘metal B’ in DNA polymerases, and indicating the readiness of the active site to proceed with the dATP insertion. The template oxoG base assumes the *syn* conformation about the glycosidic bond and uses its Hoogsteen edge to pair with the incoming dATP (Fig. 2b, c). Such arrangement is evident from the well-ordered electron density map for both residues of the pair (Fig. 2c) and is observed in both types of the PrimPol oxoG•dATP complexes. Thus, the protonated N7 atom of the oxoG residue forms a hydrogen bond with the N1 of the dATP (acceptor) and the *O*^*6*^ atom of the oxoG (acceptor) interacts with the *N*^*6*^ of the dATP (donor). The C1′−C1′ distance in this oxoG*(syn)*•dATP base pair is 10.6 Å, allowing for a perfect fit into the active site of PrimPol. There is also a stabilizing intramolecular bond within the oxoG*(syn)* residue, between the *N*^*2*^ atom of the base and the oxygen atom of its phosphate group.

While the oxoG*(syn)* residue fits the PrimPol active site without altering any active site residues (Fig. 2b) or template strand backbone conformation (Fig. 2c), the efficiency of dATP incorporation opposite the oxoG is markedly reduced compared to that of dCTP, namely 5.7-fold less in the presence of Mg^2+^ and 1.8-fold less in the presence of Mn^2+ 22^. From the structure, this reduction in incorporation of dATP versus dCTP is not caused by any structural interference per se; instead, it may reflect the reduced thermodynamic stability of an oxoG•dATP Hoogsteen base-pair compared to an oxoG-dCTP WC base-pair^43^.

### Extension from the correct C opposite oxoG

**Fig. 3:**
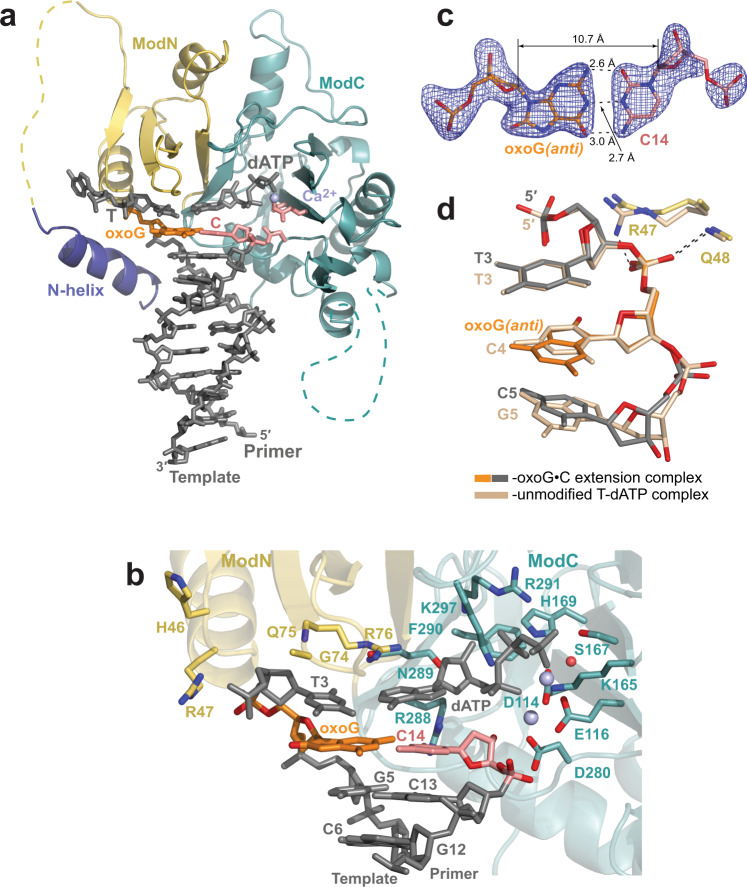
Structure of human PrimPol extending from a correct primer C base opposite the oxoG template base. **a** The overall structure of the ternary PrimPol oxoG•C extension complex (molecule A of the asymmetric unit, AU). The N-helix and modules ModN and ModC are shown in cartoon representation in dark blue, yellow, and cyan, respectively. The DNA is shown as gray sticks, the Ca^2+^ ion (‘metal B’) is shown as a light blue sphere. The oxoG residue (orange) is forming a base pair with the primer terminal C base (red). The incoming dATP forms a nascent base pair with a template T base, which is 5′-adjacent to the oxoG residue. The unstructured regions in the ModN and ModC modules are shown as dashed yellow and cyan lines, respectively. The side chains of key catalytic active-site residues Asp114, Glu116, and Asp280 are shown as red sticks. **b** A close-up view of the oxoG template base and the 3′-deoxy primer C14 base in the PrimPol active site (molecule B in the AU). There are two Ca^2+^ ions (metal ‘A’ and ‘B’) in this complex shown as light blue spheres, and the water molecule coordinated by the Ca^2+^ ion is shown as a small red sphere. The key catalytic residues (Asp114, Glu116, and Asp280), the residues contacting the incoming dATP (Lys165, Ser167, His169, Arg288, Asn289, Phe290, Arg291, and Lys297), the templating base (Gly74, Gln75, and Arg76), and the rest of the template strand (Lys10, His46, and Arg47) are shown in sticks and with oxygen atoms in bright red and nitrogen atoms in blue. **c** A simulated annealing Fo−Fc omit map (contoured at 2.5σ-level at 2.10 Å resolution and colored in blue) showing the clear electron density for the entire oxoG residue in the *anti* conformation and its partner C14 residue. Hydrogen bonds are indicated by black dashes. **d** Similar phosphate backbone conformations of T3-oxoG4*(anti)*-C5 (gray and orange) and unmodified T3-C4-G5 (beige) template strand segments within the PrimPol active site of their respective ternary complexes. The complexes are superimposed by ModN and ModC of PrimPol protein. The main-chain nitrogen atom of Gln48 contacts the oxygen atom of the phosphate group of the oxoG*(anti),* similarly to its contact with the phosphate group of the C4 in the unmodified complex. The guanidinium group of Arg47 of ModN, however, repositions to contact a neighboring protein chain in the crystal (main-chain oxygen atom of Gly124 of a symmetry-related molecule, not shown).

The 2.1 Å resolution structure of PrimPol, extending a correct primer C opposite the oxoG, depicts the next step in accurate bypass of this oxidative lesion (Fig. 3a). The structure of the extension complex is almost identical to the unmodified complex (rmsd of ~0.46 Å over 249 Cαs of molecule A, for example; Supplementary Fig. 5).

In the oxoG•C extension complex, PrimPol forms the next correct nascent base pair (Fig. 3a, b). Thus, the T3 template base, which is 5′-adjacent to the oxoG residue, is now paired with the incoming dATP. Both ‘metal A’ and ‘B’ Ca^2+^ ions are observed in molecule B of the complex (Fig. 3b).

The oxoG*(anti)* at the extension position of the active site (Fig. 3b) is in a Watson−Crick base pair configuration with the 2′-deoxy-3′-C14 terminal primer base (Fig. 3c). Surprisingly, the conformation of the phosphate group of the oxoG is similar to that of the phosphate of an unmodified residue at the same position of the PrimPol complex (the C template strand residue in the undamaged complex) (Fig. 3d). The steric clash of the O8 atom with the sugar-phosphate backbone that arises with oxoG*(anti)* is alleviated by adjustments of the backbone torsion angles of the phosphate group and a slight rotation of the sugar ring compared to the values observed for B-DNA. Thus, the conformation of the sugar-phosphate backbone of the template base at the extension position of the PrimPol active site is preset to accommodate the oxoG*(anti)*. Consequently, the main-chain nitrogen atom of Gln48 forms a hydrogen bond with the phosphate group of either the oxoG*(anti)* or an unmodified residue in a similar manner (Fig. 3d). The guanidinium group of Arg47 of ModN, however, repositions to contact a neighboring protein chain in the crystal (main-chain oxygen atom of Gly124 of a symmetry-related molecule, not shown).

Overall, the oxoG*(anti)* template strand residue is accommodated at the extension position without significant adjustments to the active site or to the position of its partner primer terminal C residue, compared to the unmodified structure. This correlates with the near-normal efficiency extension from oxoG•C pair by PrimPol (∼75−85% of that observed for undamaged DNA)^22^.

### Extension from the incorrect A opposite oxoG

**Fig. 4:**
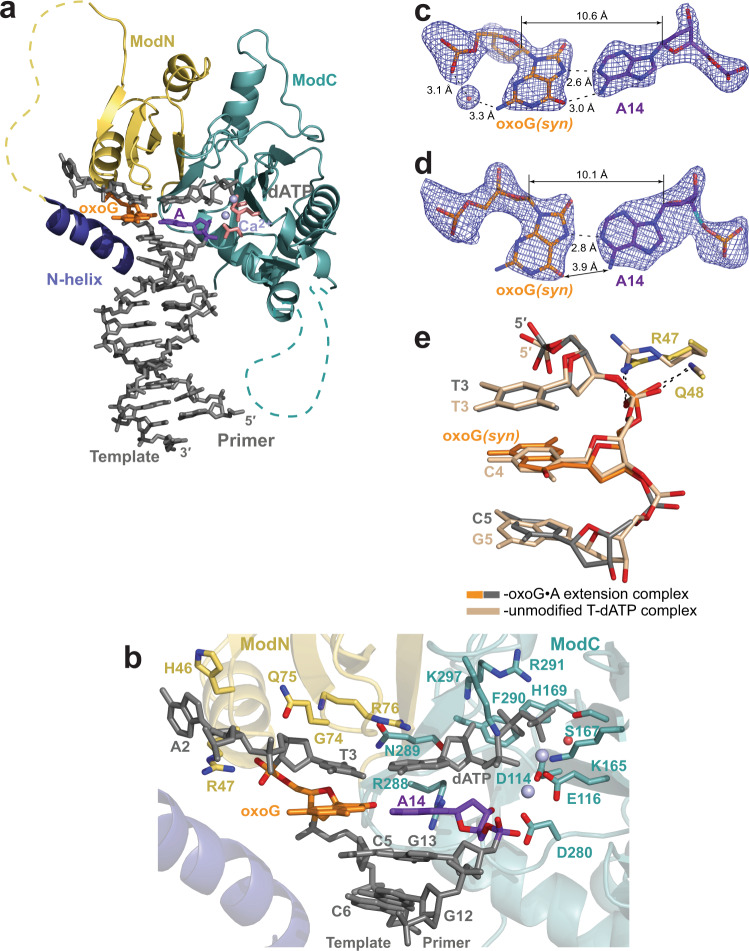
Structure of human PrimPol extending from an incorrect primer A base opposite the oxoG template base. **a** The overall structure of the ternary PrimPol oxoG•A extension complex (molecule A of the asymmetric unit, AU). The N-helix and modules ModN and ModC are shown in cartoon representation in dark blue, yellow, and cyan, respectively. The DNA is shown as gray sticks, the two Ca^2+^ ions (metals ‘A’ and ‘B’) are shown as light blue spheres. The oxoG residue (orange) is forming a base pair with the primer terminal A base (purple). The incoming dATP forms a nascent base pair with a template T base, which is 5′-adjacent to the oxoG residue. The unstructured regions in the ModN and ModC modules are shown as dashed yellow and cyan lines, respectively. The side chains of key catalytic active-site residues Asp114, Glu116, and Asp280 are shown as red sticks. **b** A close-up view of the oxoG template base and the 3′-deoxy primer C14 base in the PrimPol active site (molecule A in the AU). Two Ca^2+^ ions (metals ‘A’ and ‘B’) in this complex shown as light blue spheres, and the water molecule coordinated by the Ca^2+^ ion is shown as a small red sphere. **c** A simulated annealing Fo−Fc omit map (contoured at 2.5σ-level at 2.07 Å resolution and colored in blue) showing the clear electron density for the oxoG residue in the *syn* conformation and its partner A14 residue in molecule B of the AU. The water molecule that facilitates the intramolelecular bond between the *N*^*2*^ and the phosphate group of the oxoG residue is observed only in molecule B. **d** A simulated annealing Fo−Fc omit map (contoured at 2.5σ-level) showing the electron density for the oxoG*(syn)* and its partner A14 residues in molecule A of the AU. There is only one hydrogen bond in this base pair. **e** Similar phosphate backbone conformations of T3-oxoG4*(syn)*-C5 (gray and orange) and unmodified T3-C4-G5 (beige) template strand segments within the PrimPol active site of their respective ternary complexes. The main-chain nitrogen atom of Gln48 and the side chain of Arg47 contact the phosphate group of the oxoG*(syn)* similarly to the protein contacts with the phosphate group of C4 in the unmodified complex.

The structure of PrimPol extending from an incorrect primer A base opposite the oxoG is solved to 2.07 Å resolution (Fig. 4a and Supplementary Fig. 6). The oxoG residue is in the *syn* conformation and uses its Hoogsteen edge to form a base pair with the 3′-terminal primer base A14 (Fig. 4b). In molecule B complex in the AU, the oxoG•A mispair makes both hydrogen bonds expected for a Hoogsteen base-pair (Fig. 4c), while in molecule A the mispair is more open with the *O*^*6*^ atom of the oxoG too far to interact with the *N*^*6*^ of A14 (Fig. 4d). Interestingly, in both molecules, there is no direct intermolecular hydrogen bond between the *N*^*2*^ of the oxoG*(syn)* base and its nearest backbone oxygen atom (Fig. 4c, d). This is in contrast to the oxoG*(syn)* residue in the oxoG•dATP insertion complex (Fig. 2c), where these two atoms are closer due to a different backbone conformation. Nevertheless, there is a water molecule bridging the *N*^*2*^ and the oxygen of the phosphate group of the oxoG*(syn)* in the molecule B complex (Fig. 4c). The overall conformation of the sugar-phosphate backbone of the oxoG*(syn)* template base at the extension position is very similar to the one in the unmodified complex and does not disrupt PrimPol active site (Fig. 4e).

**Fig. 5:**
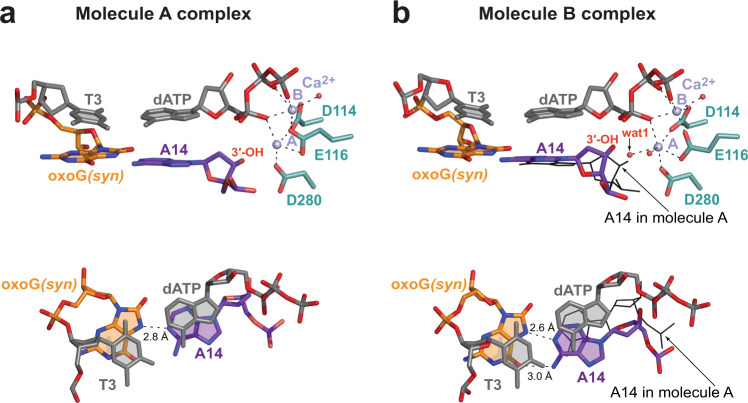
Correct and misaligned placement of the 3′-OH primer terminus in molecule A and B complexes in the asymmetric unit of the oxoG•A extension complex. **a** Molecule A complex. Top panel: the side view parallel to the DNA duplex axis. Two Ca^2+^ ions (metals ‘A’ and ‘B’) shown as light blue spheres, and the water molecule coordinated by the Ca^2+^ ion is shown as a small red sphere. Bottom panel: the view perpendicular to the DNA duplex axis, highlighting the relative positions of dATP and the 3′-primer terminal A14. **b** Molecule B complex. Top and Bottom panels as described above. The placement of A14 residue of molecule A complex is shown in black lines. The A and B molecules are superimposed by PrimPol protein. The oxygen atoms of the sugar-phosphate backbone are colored in red.

Intriguingly, while ‘metal A’ and ‘metal B’ Ca^2+^ ions are in a similar configuration in molecules A and B of the complex, the position of the 3′-OH of the primer terminus varies (Fig. 5a, b, top panels). In molecule A, even though the oxoG*(syn)* mispair with A14 is somewhat open and held by only a single hydrogen bond (Figs. 4d and 5a, bottom panel), the 3′-OH of A14 is directly below the α-phosphate of the dATP, in an orientation favorable for an in-line reaction (Fig. 5a, top and bottom panels). By contrast, the more stable oxoG*(syn)*•A mispair with two hydrogen bonds in molecule B (Figs. 4c and 5b, bottom panel), is achieved by rotation of the primer A14 residue clockwise relative to the protein active site, so the A14 is able to fulfill both hydrogen bonds with oxoG*(syn)* (Fig. 5a, b, bottom panels). The rotation of the primer terminal A14 is evident by an increase of a twist angle between the base of A14 relative to the base of the incoming dATP that is positioned similarly in both molecules, from 25° in molecule A complex to 37° in the molecule B complex. Consequently, the rotation causes the 3′-OH of A14 in molecule B complex to shift away from the optimal position observed in molecule A. A new water molecule coordinated by the ‘A’ ion, labeled ‘wat 1’ on Fig. 5b top panel, takes nearly the same place as the 3′-OH in the molecule A. The displaced 3′-OH in molecule B is further away from the catalytic Ca^2+^ ion ‘A’ and thus is poorly positioned for metal ion ‘A’-supported deprotonation needed to initiate the nucleotidyl-transfer reaction^44^. Taken together, the protrusion of the oxoG*(syn)* into the major groove causes either the decreased stability of the oxoG*(syn)*•A mispair or the misalignment of the 3′-OH primer terminus of the partner A residue. These factors appear to be responsible for 1.8- (Mg^2+^-reaction condition) to 2.1-fold (Mn^2+^-reaction condition) reduction of extension efficiency from an A compare to a C base opposite the oxoG lesion^22^.

## Discussion

7,8-dihydro-8-oxoguanine (oxoG) is a ubiquitous DNA damage lesion produced due to interactions of reactive oxygen species (ROS) with nuclear and mitochondrial DNA in human cells. Here, we provide the structural basis for human archaeo-eukaryotic primase-polymerase PrimPol’s ability to efficiently bypass the oxoG lesion in a predominantly error-free manner. We show that PrimPol accommodates an oxoG lesion in the *anti* conformation at both the insertion and the extension steps of lesion bypass, without altering a catalytically-ready alignment of the active site. This allows PrimPol to conduct a proficient error-free replication through the lesion site by insertion of a correct C base opposite the oxoG*(anti)* and extension from it. Overall, the ‘minimal’ architecture of PrimPol whereby it is devoid of contacts with the major and minor grooves of the DNA as well as the sugar-phosphate backbone of the templating nucleotide^35^ is beneficial for nearly unobstructed and correct lesion bypass of oxoG.

We also find that the mutation-inducing insertion of dATP opposite the oxoG*(syn)* does not cause any significant structural hindrance within the PrimPol active site. Why then is the efficiency of dATP incorporation opposite the oxoG reduced ~6-fold compared to dCTP^22^? One factor may be the lower thermodynamic stability of an oxoG•dATP nascent base-pair relative to that of an oxoG-dCTP base-pair^43^. Also, it is possible that a conformational *anti*-*syn* equilibrium of an unpaired oxoG template residue in a binary complex might influence a selection of an incoming nucleotide. For example, the template oxoG can exist in both conformations within the active site of X-family Pol *β*, but only the oxoG*(syn)* conformation is stabilized via hydrogen bonding interactions, and thereby promoting the binding and insertion of dATP^45^. Since PrimPol is devoid of amino acids interacting with the templating base, oxoG*(anti)* may be favored over oxoG*(syn)* due to stacking interactions with the neighboring 3′-template base, leading to the preferred incorporation of dCTP over dATP.

PrimPol is also reduced in its efficiency (~2-fold) in extending DNA synthesis from an A compared to a C base opposite the oxoG lesion^22^. We show here that during the extension step, compared to C opposite oxoG, incorrect A leads to significant deviations from the optimum alignment in the active site. That is, the protrusion of oxoG*(syn)* into the major groove in the extension complex causes either the opening of the oxoG*(syn)*-A pair with a loss of one hydrogen bond between the bases or the misalignment of the 3′-OH primer terminus when the partner A residue rotates to reestablish both hydrogen bonds within the pair. Together, these structural deviations may underlie the reduced efficiency of PrimPol in extending DNA synthesis from a 3’-terminal A base opposite an oxoG.

The PrimPol structures also shed additional light on the active-site geometry. In the unmodified PrimPol structure^35^ we observed only one Ca^2+^ ‘ion B’ chelated by the oxygen atoms of the triphosphate tail of the incoming dATP as assumed due to the use of 2′,3′-dideoxy primer terminus. Interestingly, in the current study, with the ‘native’ 3′-OH-2′-deoxy primers, a single Ca^2+^ ‘ion B’ is detected in both complex molecules of the asymmetric unit (AU) of the oxoG•dCTP insertion complex and in molecules ‘A’ of the oxoG•dATP insertion and the oxoG•C extension complexes. Thus, binding of dNTP together with ‘ion B’ to PrimPol-DNA appears to be the first step in ternary PrimPol complex assembly, similar to that observed for Y-family human Pol η by time-resolved X-ray crystallography^46,47^. In the PrimPol-DNA-dNTP-‘ion B’ complexes, the distances of primer 3′-OH to α-phosphate (Pα) of the incoming dNTP vary from 4.29 to 4.70 Å. Unexpectedly, the entrance of the second Ca^2+^ ‘catalytic ion A’ detected in the extension oxoG•C molecule B complex and oxoG•A molecule A complex change the 3′-OH to Pα distances only slightly to 3.99 and 4.11 Å, respectively. Such 3′-OH to Pα distances are at least ~0.7 Å too long for phosphoryl transfer to occur. This is different compared to Pol η, where the ternary complex with a single Ca^2+^ ‘ion B’ has the 3′-OH to Pα distance of 4.20 Å, which upon the binding of the second ion (Mg^2+^) at the catalytic ‘site A’ and exchange of ‘ion B’ to Mg^2+^ decreases to ~3.30 Å and then is further reduced by 0.5 Å during the nucleotidyl-transfer reaction^46^. Thus, the active site of PrimPol, even after the binding of both ions, appears to be less tightly organized than Pol η’s and possibly more dependent on slow thermal motion to align the catalytic groups. The slow catalytic tuning might represent the ‘rate-limiting conformational transition step’ detected for PrimPol during a pre-steady-state kinetics study^48^.

High amounts of ROS^14^ and limited repair capabilities^26,27^ make mitochondrial DNA especially vulnerable to oxidative damage^15^. Mitochondrial replicative Pol *γ* is strongly inhibited by the oxoG lesion and prefers to extend from the oxoG•A base pair^11,42^, and thus is unlikely to be involved in error-free oxoG bypass in vivo. PrimPol, may partake in that role. It is the only TLS DNA polymerase known to localize to mitochondria and is able to efficiently and mostly accurately bypass the oxoG lesions^21–25^. While PrimPol does not appear to rescue in vitro reconstituted mitochondrial replisome stalled at the oxoG lesion site^42^, it is likely that additional accessory proteins and different biochemical conditions within mitochondria in vivo might facilitate the bypass efficiency.

Taken together, PrimPol has emerged as a unique primase-polymerase that can partake in direct TLS of small DNA lesions such as oxoG. We provide here the structural underpinnings for how PrimPol is able to conduct proficient and mostly error-free oxoG lesion bypass.

## Methods

### Protein purification

The catalytic domain of human PrimPol (residues 1−354) was expressed in *Saccharomyces cerevisiae* as an N-terminally tagged glutathione S-transferase (GST) fusion protein and then purified as described previously^35^. The yeast cell pellets were resuspended in lysis buffer (50 mM TRIS-HCl (tris(hydroxymethyl)aminomethane) pH 8.0, 10% sucrose, 150 mM NaCl, 175 mM ammonia sulfate, 1 mM EDTA, 10 mM *β*-mercaptoethanol supplemented with protease inhibitors, 1 mg/mL leupeptin, 5 mg/mL chymostatin, 1 mg/mL aprotinin, 1 mg/mL pepstatin, and 0.078 mg/mL benzamidine) and lysed with a bead beater. Total cellular proteins were precipitated by ammonium sulfate, resuspended, and loaded onto a glutathione-Sepharose gravity flow column in buffer A (50 mM TRIS-HCl pH 7.5, 250 mM NaCl, 10% glycerol, 5 mM DTT). The GST-PrimPol fusion protein bound to the resin was washed with buffer A, buffer A supplemented with a total of 1 M NaCl, and again with buffer A. PrimPol protein (carrying an extra GPGGDPH peptide on its N terminus due to the expression construct design) was eluted with buffer A after on-column overnight cleavage of the GST tag with PreScission protease. PrimPol protein was then further purified on HiTrap Heparin column (GE Healthcare). The binding buffer for the Heparin column contained 25 mM TRIS-HCl (pH 7.5), 150 mM NaCl, and 2 mM DTT, and the elution buffer consisted of 25 mM TRIS-HCl (pH 7.5), 1 M NaCl, and 2 mM DTT. Finally, PrimPol protein was purified by gel-filtration on Superdex 75 column (GE Healthcare) in 25 mM TRIS (pH 7.5), 250 mM NaCl, and 2 mM tris(2-carboxyethyl) phosphate (TCEP), concentrated to ~35 mg/ml, and stored in aliquots at −80 °C.

### Crystallization

The crystals of the ternary complex with the correct C incoming nucleotide triphosphate (dCTP) opposite the template oxoG residue were obtained by incubating the human PrimPol catalytic core with a 17-nucleotide (nt) DNA template (5′-CA(oxoG)CGCTACCACACCCC-3′) (TriLink Biotechnologies Inc.) annealed to a 2′-deoxy-3′ terminated 12 nt DNA primer (5′-GGTGTGGTAGCG-3′) (Glen Research, Inc) (Supplementary Table 1) in a presence of dCTP. The crystal growth and harvesting were performed as described in our study of PrimPol structure with unmodified DNA^35^. Annealed template-primer DNA was mixed with PrimPol in a 1.2:1 molar ratio to a final concentration of 0.3 mM in 25 mM TRIS-HCl (pH 7.5), 155 mM NaCl, 1.5 mM TCEP, 12 mM CaCl_2_, and 7 mM dCTP. The complexes were incubated at room temperature for 5 min and then centrifuged at 9,300 ×*g* for 10 min at 4 °C. The crystals were grown at 20 °C by a hanging drop method against a reservoir solution containing 225−250 mM CaCl_2_ and 16−19% PEG 3350. To produce bigger crystals (rod-like, up to ~0.2 mm in length), a round of microseeding was performed. The crystals were cryoprotected stepwise in reservoir solution supplemented with 24% PEG 3350 and 10% and then 20% glycerol and flash-frozen in liquid nitrogen for X-ray data collection.

The crystals of the PrimPol ternary complex with incoming dATP opposite the template oxoG were produced with the aforementioned 17 nt template and 2′-deoxy- or 2′,3′-dideoxy-3′ terminated 13 nt DNA primer, namely 5′-GGGTGTGGTAGCG-3′ (Glen Research, Inc) or 5-GGGTGTGGTAGCG_dd_-3′ (W.M. Keck Foundation, Yale). The extension ternary complexes were obtained with a 17 nt DNA template (5′-CAT(oxoG)CCTACCACACCCC-3′), a 13 nt DNA primers with either a C or an A 2′-deoxy 3′-terminal bases (5′-GGGTGTGGTAGGX-3′, where X is C or A), and the next correct incoming base dATP. The crystals were obtained as described above for the oxoG•C complex.

### Structure determination and refinement

The X-ray diffraction data were collected at the 24-ID NE-CAT beamline at Advanced Photon Source in Chicago and were processed by the RAPD pipeline (http://necat.chem.cornell.edu/). We solved the structure of the PrimPol ternary complex with the correct incoming dCTP opposite the template oxoG base (oxoG•dCTP) by the molecular replacement (MR) method (Phaser)^49^ in the Phenix program package^50^ using the PrimPol ternary complex structure with the unmodified DNA (PDB ID: 5L2X)^35^ as a search model. The model building was finished manually in Coot^51^ based on the electron density maps calculated in PHENIX Refine^50^. The final model of the oxoG•dCTP complex was refined in PHENIX Refine to 2.6 Å resolution and belongs to P1 space group with unit cell dimensions of *a* = 52.6 Å, *b* = 65.7 Å, *c* = 75.2 Å, *α* = 69.7°, *β* = 82.8°, and *γ* = 88.5°. The structure is refined to *R*_free_ of 28.6% and *R*_work_ of 23.9% and consists of two PrimPol molecules, two DNA template-primer molecules, two dCTP, two Ca^2+^ ions, and a total of 92 solvent molecules. The placement and conformation of the oxoG and dCTP residues was verified using simulated annealing omit maps calculated in PHENIX^50^ with the residues omitted from the model before heating to 2,000 K and then slowly cooling. The average B-factor for the portion of the DNA interacting with PrimPol protein via the sugar-phosphate backbone (residues oxoG3-C4-G5-C6 of the template and G14 of the primer strands) is lower (68.9 Å^2^) than the overall average B-factor for the DNA (115.3 Å^2^) and is similar to the average B-factor of the protein (64.4 Å^2^). The relatively high B-factors for ‘unbound’ portions of the DNA reflect their conformational flexibility in the absence of direct contact with PrimPol.

The crystals with the incoming dATP opposite the template oxoG and containing 2′,3′-dideoxy-3′ terminated 13 nt DNA primer, diffracted to 2.37 Å resolution and belong to P1 space group with the unit cell parameters similar to the oxoG•C complex. We have used the oxoG•dCTP complex as a search model to solve the structure by MR. The oxoG•dATP structure is refined to *R*_free_ of 27.7% and *R*_work_ of 22.0% and consists of two PrimPol molecules, two DNA template-primer molecules, two dATPs, three Ca^2+^ ions, and a total of 106 solvent molecules.

The structure of the complex with the incoming dATP opposite the template oxoG and containing 3′-deoxy-3′ terminated 13 nt DNA primer, was refined to 2.58 Å resolution, also in P1 space group with similar unit cell dimentions. The structure is refined to *R*_free_ of 30.7% and *R*_work_ of 24.8% and consists of two PrimPol molecules, two DNA template-primer molecules, two dATPs, three Ca^2+^ ions, and 24 water molecules. Similar to the oxoG•dCTP complex, in the oxoG•dATP structures, the average B-factors for the portions of the DNA interacting with PrimPol protein are comparable to that of the protein, whereas B-factors for the regions of the DNA that do not interact with PrimPol are higher, reflecting their greater conformational freedom.

The oxoG•C PrimPol extension complex structure with a correct primer C opposite the template oxoG and the next correct incoming nucleotide (dATP) was also determined by MR. Because the unit cell dimensions of the complex were isomorphous to the unit cell dimensions of the parent structure with undamaged DNA (5L2X), we imported the R-free set from 5L2X using the Phenix reflection file editor. We also conducted simulated annealing on the coordinates after MR, and rebuilt the model manually in Coot^51^ using the electron density maps calculated in PHENIX Refine^50^. The oxoG•C structure was refined to 2.1 Å resolution with several cycles of TLS refinement conducted at the end of the refinement process. After TLS refinement, *R*_free_ and *R*_work_ are 21.3 and 18.7%, respectively, and the final model consists of two PrimPol molecules, two DNA template-primer molecules, two dATPs, four Ca^2+^ ions, and a total of 162 solvent molecules. The oxoG•A PrimPol extension complex structure with an incorrect primer A opposite the template oxoG and the next incoming nucleotide (dATP) was similarly determined by MR with 5L2X as a search model. Again, because the unit cell dimensions of the complex were very close to the unit cell dimensions of 5L2X, we imported the R-free set from the parent structure and followed the refinement process described above for the oxoG•C extension complex, to a resolution of 2.07 Å. After TLS refinement, the *R*_free_ and *R*_work_ are 21.9 and 20.1%, respectively. The final model consists of two PrimPols, two DNA template-primer molecules, two dATP, four Ca^2+^ ions, and a total of 131 solvent molecules. In the extension complexes, the B-factors for the protein-bound DNA nucleotides are lower than for the DNA overall, and are closer in values to the protein residues.

Curiously, the validation reports calculated upon the deposition of data to Protein Data Bank (PDB), indicate that the extension complex structures have a somewhat high number of RSRZ outlier protein residues (14.0% in the oxoG•C and 13.0% in the oxoG•A). However, most of the flagged residues have an unequivocally good fit to the electron density map. For example, in the oxoG•C complex structure, residues Phe290 in chain A flagged with RSRZ = 4.5 and in chain B flagged with RSRZ = 4.8, have well-defined densities that are equivalent in quality to the densities for the not-flagged Phe166A and Phe166B residues (Supplementary Fig. 7). In addition, Phenix comprehensive validation, indicates that both Phe290A and Phe290B have a real space correlation coefficient (CC) of 0.98 and 0.99, respectively.

The crystal data, together with the data collection and refinement statistics, are summarized in Table 1. Figures were prepared using PyMOL^52^ (The PyMOL Molecular Graphics System, Version 2.0 Schrödinger, LLC).

### Reporting summary

Further information on experimental design is available in the Nature Research Reporting Summary linked to this paper.

## Supplementary information

Supplementary Information.

Reporting summary

## Data Availability

Atomic coordinates and structure factors have been deposited in the Protein Data Bank (PDB) under accession codes 7JK1, 7JKL, 7JKP, 7JL8 and 7JLG. Other data are available from the corresponding authors upon reasonable request.
