# Supplementary material for: Structural basis of DNA synthesis opposite 8-oxoguanine by human PrimPol primase-polymerase
Source: Nat Commun. 2021 Jun 29;12:4020. doi: 10.1038/s41467-021-24317-z (PMC8241999; doi:10.1038/s41467-021-24317-z)
Supplement: Supplementary file 1 — Supplementary Information. [file 41467_2021_24317_MOESM1_ESM.pdf]

Supplementary Information

Structural basis of DNA synthesis opposite 8-oxoguanine by human PrimPol primase-polymerase

Olga Rechkoblit[1]*, Robert E.Johnson[2], Yogesh K. Gupta[1,3], Louise Prakash[2], Satya Prakash[2], and Aneel K. Aggarwal[1]*

[1]Department of Pharmacological Sciences
Icahn School of Medicine at Mount Sinai
New York, NY, USA

[2]Department of Biochemistry and Molecular Biology
University of Texas Medical Branch
Galveston, TX, USA

[3]Present address:
Greehey Children's Cancer Research Institute &
Department of Biochemistry and Structural Biology
University of Texas Health at San Antonio
San Antonio, TX, USA

*Correspondence: olga.rechkoblit@mssm.edu (O.R.),
aneel.agarwal@mssm.edu (A.K.A.)

Supplementary Notes 1-2
Supplementary Figures 1-7 and **Supplementary Figure Legends**
Supplementary Table 1
Supplementary References

Supplementary Note 1. DNA-DNA crystal packing interactions in the oxoG•dCTP insertion PrimPol ternary complex

To obtain crystals of PrimPol in a ternary complex inserting dCTP opposite the template oxoG lesion we have used a 17-nucleotide (nt) DNA template (5′–CA(oxoG)CGCTACCACACCCC–3′) and DNA primers of different lengths. The best diffracting crystals were produced with a 2′-deoxy 3′-terminated 12 nt DNA primer (5′–GGTGTGGTAGCG–3′) that leaves the last two nucleotides of the template strand from its 3′-end unpaired. The ordered electron density for the PrimPol-unbound end of the DNA duplex let us now observe the extensive end-to-end DNA-DNA duplex interactions promoting the crystal formation (Supplementary Fig. **2**) and rationalize our experimental design. Unexpectedly, the end-to-end DNA-DNA duplex crystal packing interactions (Supplementary Fig. **2a**) produce a triple strand DNA helix involving four CGC^+ triads formed by the template 3′-terminal C14-C15-C16-C17 and the primer 5′-end G3-G4 bases of the two duplexes (Supplementary Fig. **2b-d**).

To form a CGC^+ triad, the third C^+ base (the C base with a protonated N3 atom) invades a GC Watson-Crick pair from the major groove edge and forms hydrogen bonds with the Hoogsteen edge of the G base[1]. Thus, the two hydrogen bonds are formed between the O^6 atom of the G base (acceptor) and the N^4 amino group of the C^+ base (donor) and between the N7 atom of the G base (acceptor) and the protonated N3 atom of the C^+ base (donor). Hence, in a continuous CGC^+ type of a triple helix, the third single-stranded C-rich oligonucleotide (C^+-strand) binds to the major groove of a duplex DNA and interacts with the G-rich sequence through Hoogsteen base-pairing in a parallel (forward Hoogsteen) alignment with respect to the G-rich strand orientation.

Notably, the stack of the four CGC^+ triads produced in the oxoG•dCTP PrimPol crystal between the two DNA duplexes with 2 nts 3′-overhangs is unusual, because it formed by two oppositely oriented two-triad segments (Supplementary Fig. **2b-d**). Peculiar, the invading C^+ entity is not continuous and consists of the two segments, the 3′-C17-C16-5′ template strand nts of the molecule B of the crystallographic asymmetric unit (AU) followed by 5′-C16-C17-3′ template strand segment of the molecule A of the AU running in the opposite direction. The polypurine G-rich oligonucleotide, thus, also consists of the two segments running in the opposite direction, 3′-G4-G3-5′ segment of molecule A primer strand and 5′-G3-G4-3′ segment of molecule B primer strand. Consequently, the

two segments comprising the Watson-Crick interacting C nts are running in the opposite directions and involve 5′-C14-C15-3′ of molecule A template strand and 3′-C15-C14-5′ of molecule B template strand.

Altogether, the placement of two 5′-CC-3′/3’-GG-5′ base pairs followed by the 5′-CC-3′ sticky end leads to the end-to-end DNA stacking of two two-tier triplexes. Such DNA design can be exploited for crystal growth of other DNA-binding proteins.

Supplementary Note 2. DNA-DNA crystal packing interactions in the oxoG•dATP insertion PrimPol ternary complex

To capture PrimPol misinserting dATP opposite the oxoG lesion, we obtained crystals with the aforementioned 17-mer template and longer, 13 nt DNA primer, (5′–GGGTGTGGTAGCG–3′). The 13 nt primer produces a single 3′-C template base overhang. This DNA template-primer generates distinct from the oxoG•dCTP complex packing interactions and produces a conventional continuous triple strand CGC^{+}-type DNA helix[1] (Supplementary Fig. **4a**). Thus, the molecule A template strand 3′-end 5′-C14-C15-C16-3′ nts form the C-strand (the density for C17 residue is poorly ordered). The C-strand interacts through its Watson-Crick edges with the complimentary primer 5′-end 3′-G4-G3-G2-5′ nts of molecule A that comprise the G-rich strand (Supplementary Fig. **4b-d**). The invading third C^{+} strand consists of 3′-C17-C16-C15-5′ nts of the molecule B template strand and runs in a direction parallel to the G-strand and forms Hoogsteen base pair with it.

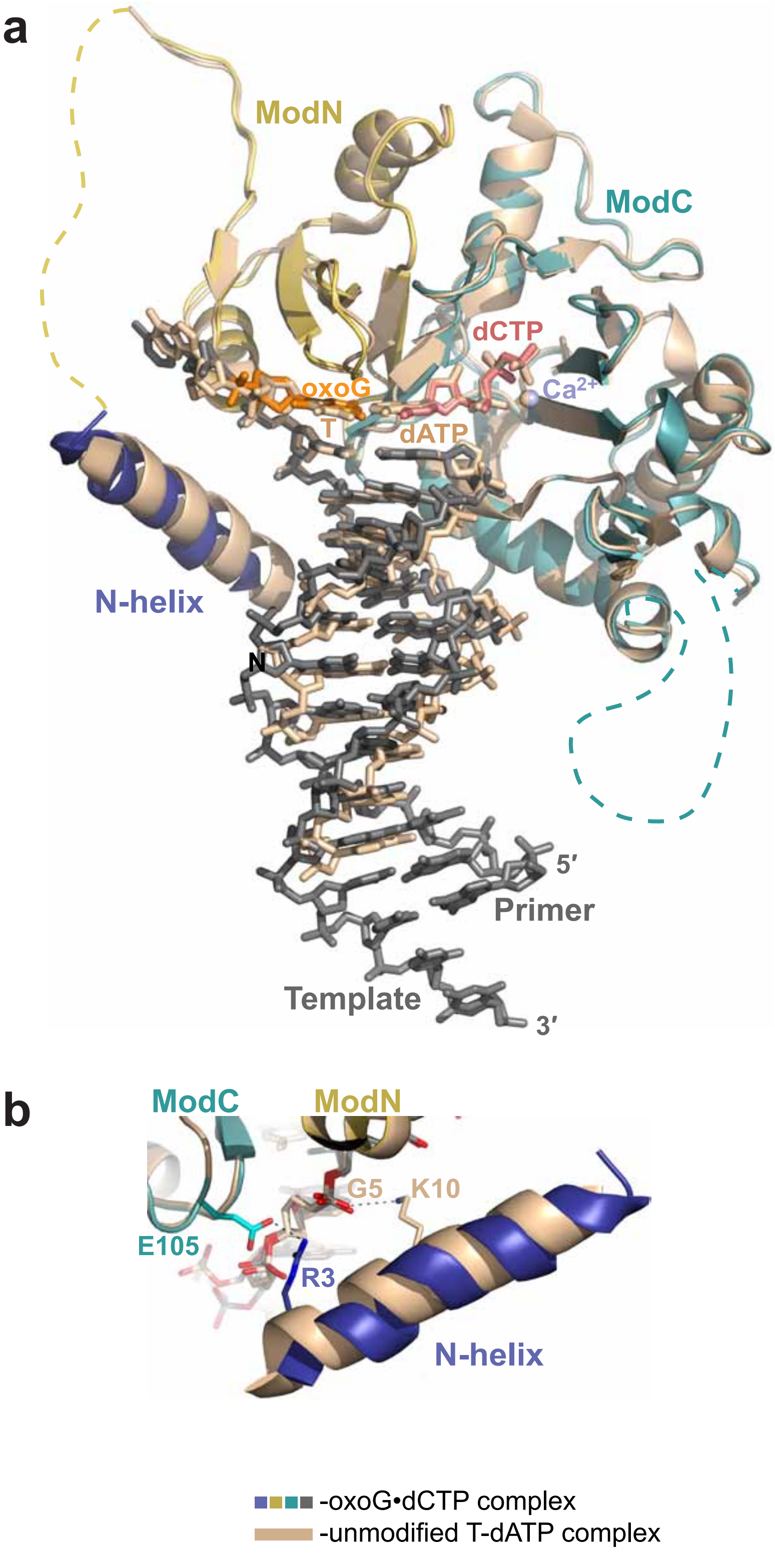

Supplementary Figure 1

Supplementary Figure 1. Comparison of the oxoG•dCTP and the unmodified T-dATP PrimPol ternary complexes. a Superposition of the oxoG•dCTP and the unmodified T-dATP PrimPol ternary complexes. Both complexes are shown in cartoon representation and the DNA is shown as sticks. The oxoG•dCTP PrimPol ternary complex is shown in colors: the N-helix in dark blue, modules ModN in yellow and ModC in cyan, the DNA in gray, and the Ca^{2+} ion is shown as a light blue sphere. The oxoG residue is shown in orange and the incoming dCTP is in red. The entire unmodified T-dATP complex (PDB ID: 5L2X)[2] is colored in beige. The unstructured regions in the ModN and ModC modules are shown as dashed lines. The complexes are superimposed by the ModN and ModC of PrimPol protein. Though the overall protein structures in the two complexes are almost identical (Cα rmsd of 0.35 Å), the N-helix of the oxoG•dCTP complex is slightly shifted away from the DNA duplex (~2.5−3 Å shift and ~3° rotation). The 17 nucleotide (nt) long template and the 13 nt primer are fully ordered in the oxoG-containing complex. In the unmodified complex the free (polymerase unbound) end of the template-primer DNA duplex is partially disordered. The disordered DNA portion in the unmodified complex includes the last four nucleotides from the 3′ end of the template strand and the last two 5′ nucleotides of the primer strand. **b** A close up view of the N-helix in the oxoG•dCTP and the unmodified complex. In the oxoG-dCTP complex there is a salt bridge between the side chains of Arg3 of the N-helix and Glu105, the last C-terminal residue of ModN. Arg3 side chain is disordered in the unmodified complex. However, in the unmodified complex, Lys10 forms a hydrogen bond with the oxygen atom of the phosphate group G5; the side chain of Lys10 is disordered in the oxoG-dCTP complex. The DNA backbone oxygen atoms are colored in red.

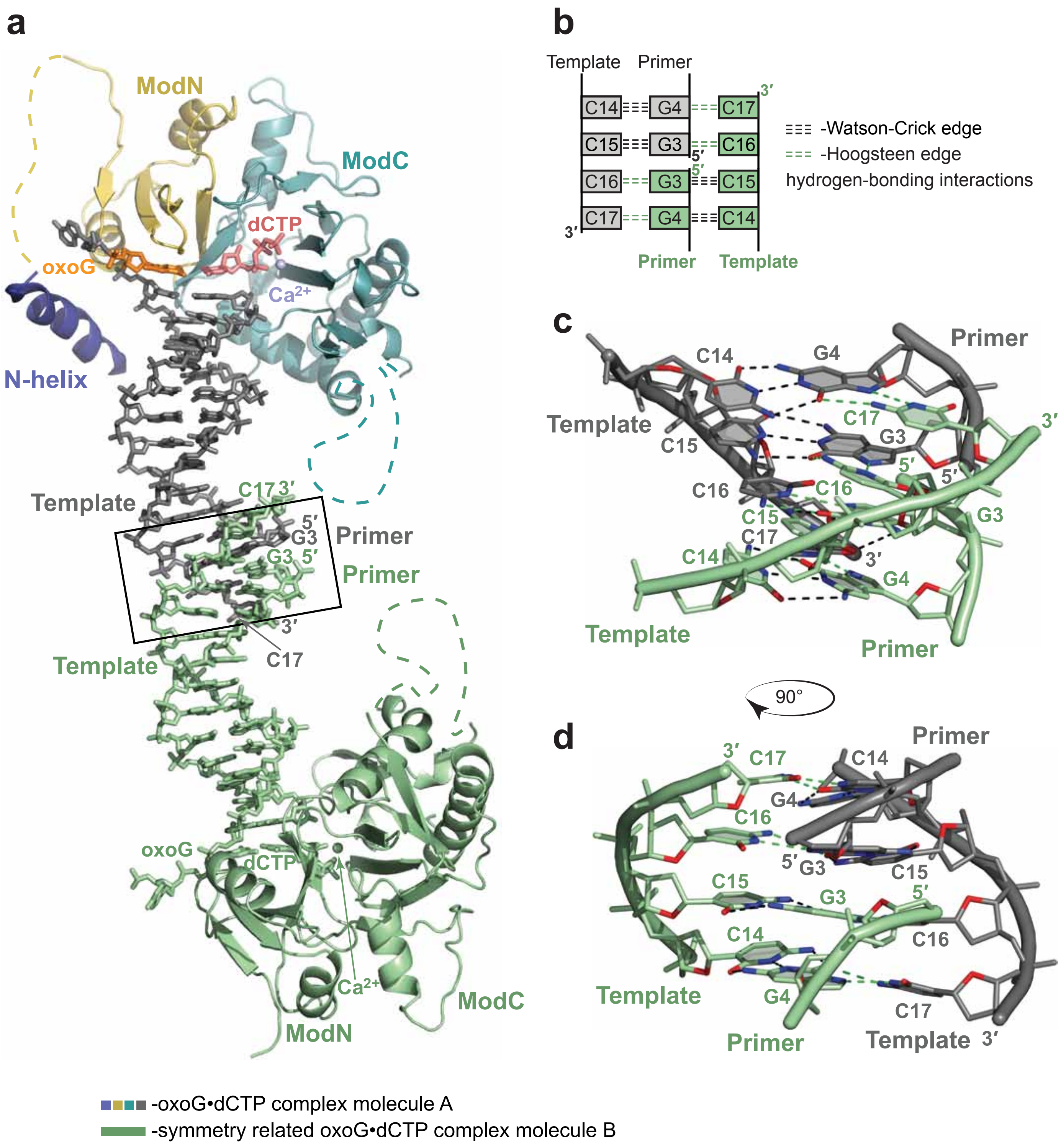

Supplementary Figure 2

Supplementary Figure 2. Crystal packing interactions in the oxoG•dCTP complex crystals. a The overall view of the oxoG•dCTP crystal packing. The two ternary complexes (molecules A and B) in the crystallographic asymmetric unit form end-to-end DNA-DNA duplex interactions that favorably promote crystal formation and growth. The oxoG•dCTP PrimPol ternary complex A is shown in colors: the N-helix in dark blue, modules ModN in yellow and ModC in cyan, the DNA in gray, and the Ca^{2+} ion is shown as a light blue sphere. The oxoG residue is shown in orange and the incoming dCTP is in red. The symmetry related complex B molecule is colored in green. Residues 1 to 17 of the N-helix are apparently disordered in complex B similar to what was observed for the B complex in the unmodified structure[2]. The bases involved in the duplex-duplex interactions (C14, C15, C16 and C17 of the template strand and G3 and G4 of the primer strand of the molecules A and B) are highlighted with the rectangular frame. **b** The schematics of the triple strand DNA helix interactions between the two duplexes. The Watson–Crick pairing interactions between the C and G bases are shown by black dash lines, the interactions between the C and the Hoogsteen edge of G are shown by green dash lines. **c** The view of the triple DNA helix formed in the crystal from the major groove side of the molecule A duplex. The triple helix consists of the four CGC^+ triads. The molecule A C14 template base and its Watson-Crick edge-pairing partner G4 of the primer strand form the 'top' triad with the template C17 base of molecule B. This molecule B C17 base invades the molecule A's duplex from the major groove edge and forms hydrogen bonds with the Hoogsteen edge of the G4 base. Thus, the two hydrogen bonds are formed between the O^6 atom of the G4 base (acceptor) and the N^4 amino group of the C17 base (donor) and between the N7 atom of the G4 base (acceptor) and the most likely protonated N3 atom of the C17 (donor) base. The oxygen atoms and the nitrogen atoms of the bases that are involved in base pairing hydrogen-bonding interactions are colored red and blue, respectively. The O4′ atoms of the sugar moieties are colored red to highlight the directionality of the DNA strands. **d** The 90° counter-clock rotated view of panel **c**. This view provides a clearer depiction of the two 'bottom' triads.

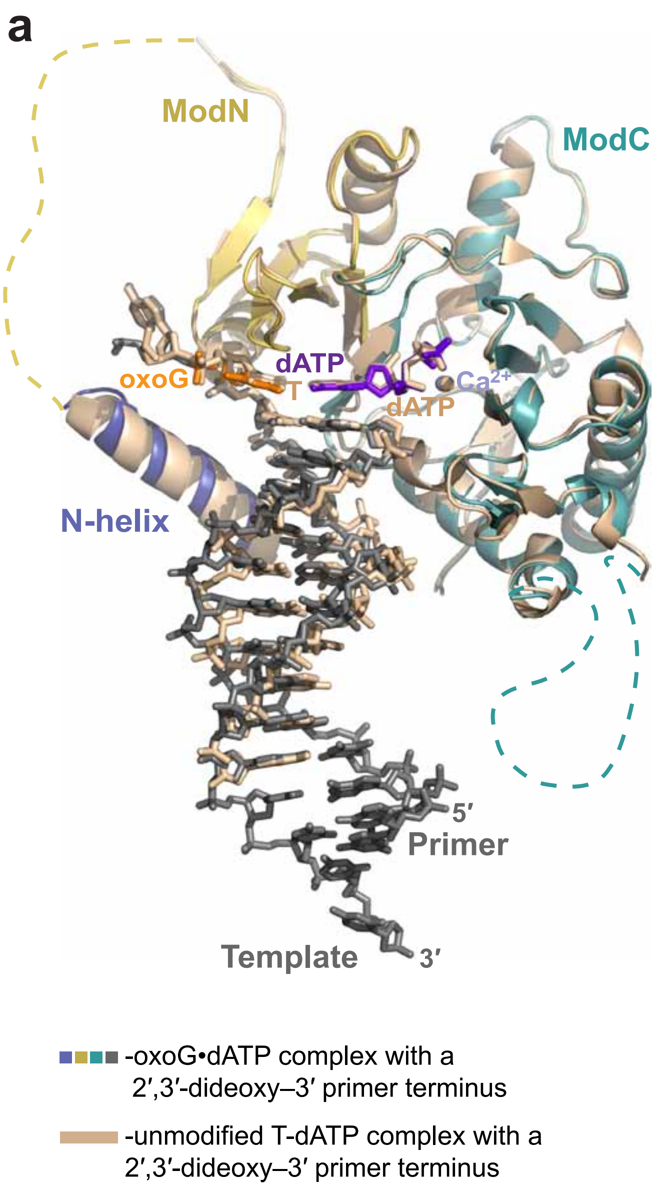

Supplementary Figure 3

Supplementary Figure 3. Comparison of the oxoG•dATP and the unmodified T-dATP PrimPol ternary complexes. a Superposition of the oxoG•dATP complex with the 2′,3′-dideoxy terminated 3′-end of the primer strand and the unmodified T-dATP PrimPol ternary complex that also has the 2′,3′-dideoxy terminated 3′-end of the primer strand. Both complexes are shown in cartoon representation and the DNA is shown as sticks. The oxoG•dATP PrimPol ternary complex is shown in colors: the N-helix in dark blue, modules ModN in yellow and ModC in cyan, the DNA in gray, and the Ca^{2+} ion is shown as a light blue sphere. The oxoG residue is shown in orange and the incoming dATP is in purple. The entire unmodified T-dATP complex (PDB ID: 5L2X)[2] is colored in beige. The unstructured regions in the ModN and ModC modules are shown as dashed lines. The complexes are superimposed by the ModN and ModC of PrimPol protein. **b** Superposition of the two oxoG•dATP complexes. The complex with the 2′,3′-dideoxy terminated primer 3′-end is colored as described above and the complex with the 2′-deoxy 3′-primer end (an intact 3′-OH group) is colored in light blue. Superposition of the structures is performed as described above.

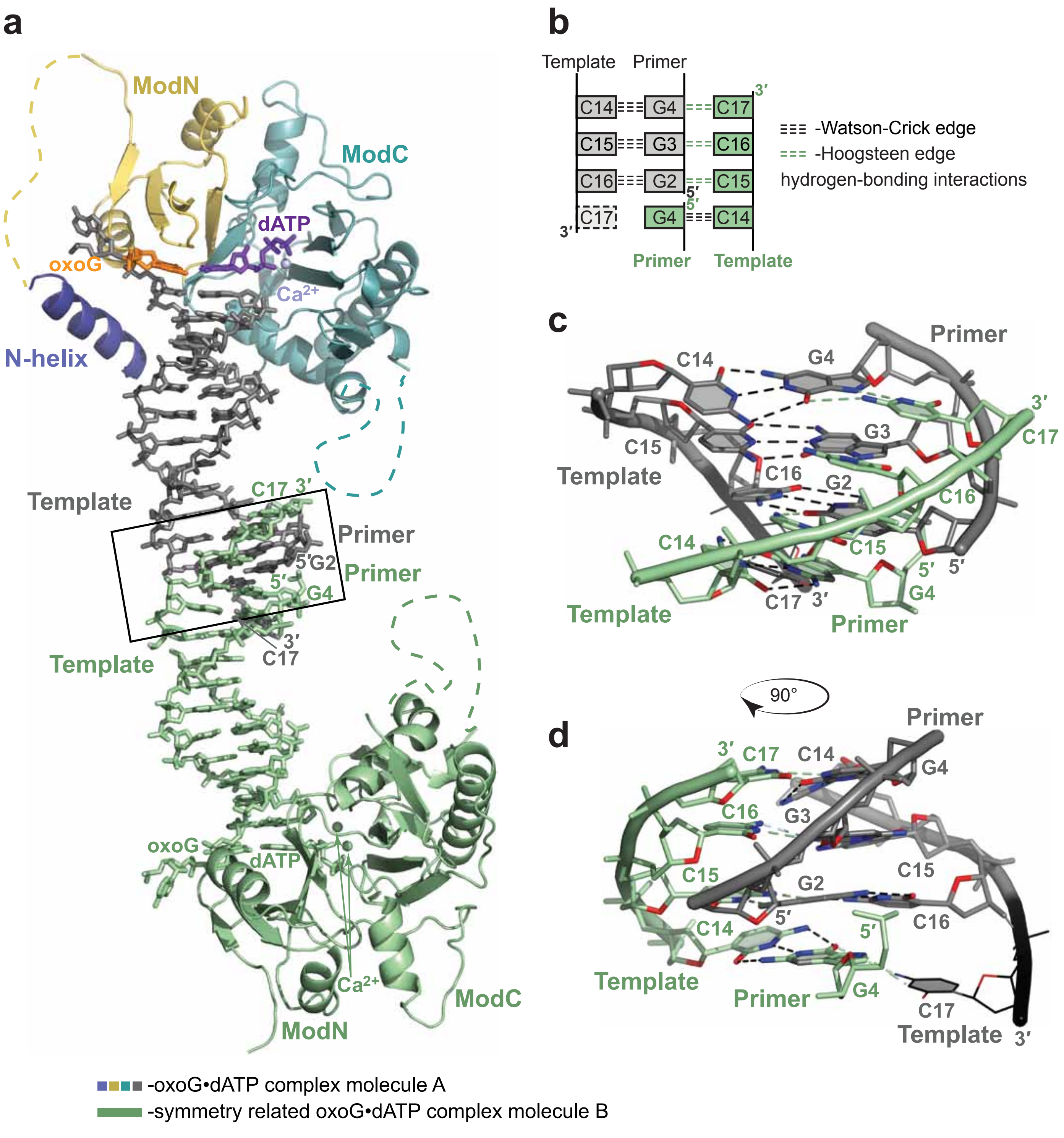

Supplementary Figure 4

Supplementary Figure 4. Crystal packing interactions in the oxoG•dATP complex crystals. a The overall view of the oxoG•dATP crystal packing. Both oxoG•dATP complexes, with the 2′,3′-dideoxy 3′-end and 2′-deoxy-3′-end of the primer strands have similar packing interactions. The higher resolution 2′,3′-dideoxy-terminated primer-containing complex is shown in the figure. The two ternary complex molecules (A and B) in the crystallographic asymmetric unit form end-to-end DNA-DNA duplex interactions that most likely favorably promote crystal formation and growth. The oxoG•dATP PrimPol ternary molecule A complex is shown in colors: the N-helix in dark blue, modules ModN in yellow and ModC in cyan, the DNA in gray, and the Ca^{2+} ion is shown as a light blue sphere. The oxoG residue is shown in orange and the incoming dCTP is in purple. The symmetry related B molecule complex is colored in green. Residues 1 to 17 of the N-helix are apparently disordered in complex B similar to what was observed for the B complex in the unmodified structure[2]. The bases involved in the duplex-duplex interactions of molecule A, namely, C14, C15, C16 of the template strand and G2, G3 and G4 of the primer strand, and of the molecule B, C17, C16, C15 are highlighted with the rectangular frame. **b** The schematics of the triple strand DNA helix formation between the two duplexes. The Watson–Crick pairing interactions between the C and G bases are shown by black dash lines, the interactions between the C and the Hoogsteen edge of G are shown by green dash lines. The electron density for molecule A template base C17 is partially disordered and the base is shown with dashed lines in the schematics. **c** The view of the triple DNA helix formed in the crystal from the major groove side of the molecule A duplex. The triple helix consists of the three CGC^{+} triads. The molecule A C14 template base and its Watson-Crick edge-pairing partner G4 of the primer strand form the “top” triad with the template C17 base of molecule B. This molecule B C17 base invades the molecule A’s duplex from the major groove edge and forms hydrogen bonds with the Hoogsteen edge of the G4 base. Thus, the two hydrogen bonds are formed between the O^6 atom of the G4 base (acceptor) and the N^4 amino group of the C17 base (donor) and between the N7 atom of the G4 base (acceptor) and the most likely protonated N3 atom of the C17 (donor) base. The oxygen atoms and the nitrogen atoms of the bases that are involved in base pairing hydrogen-bonding interactions are colored red and blue, respectively. The O4′ atoms of the sugar moieties are colored red to highlight the directionality of the DNA strands. **d** The 90° counter-clock rotated view of panel **c**. This view provides a clearer depiction of the ‘bottom’ triad.

Because the electron density for the base of C17 is partially disordered the C17 base is shown in line representation.

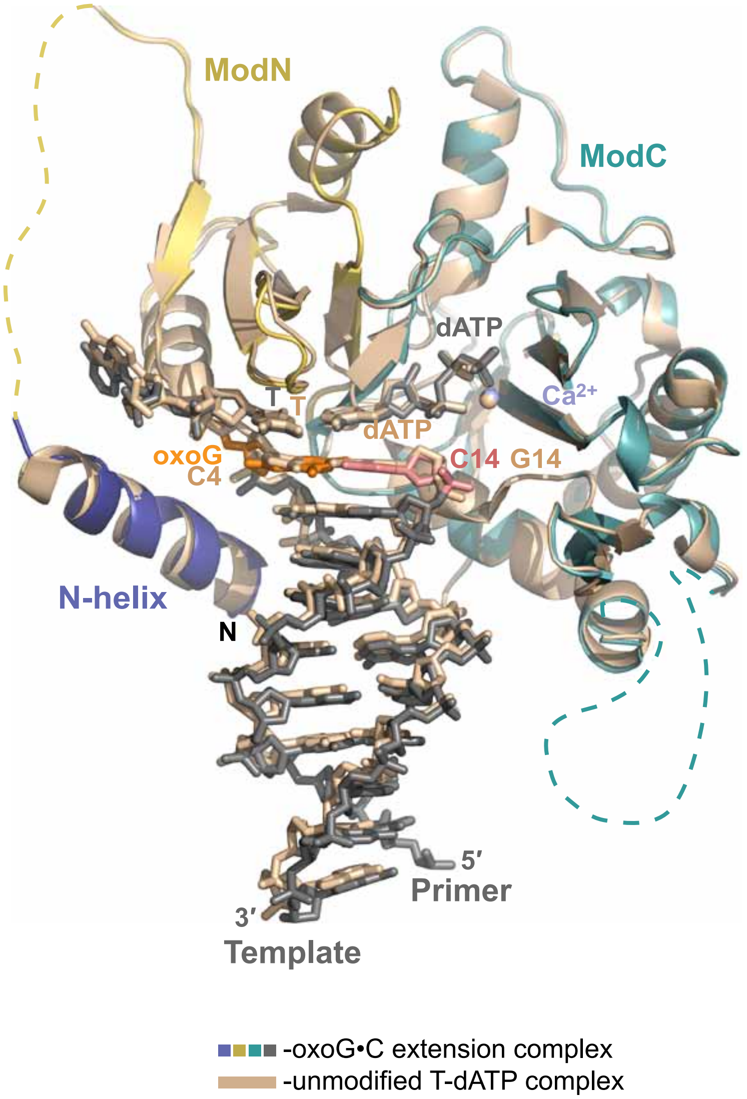

Supplementary Figure 5

Supplementary Figure 5. Comparison of the oxoG•C extension and the unmodified PrimPol ternary complexes. The oxoG•C extension complex has the 3′-deoxy terminated 3′-end of the primer C base and the unmodified T-dATP PrimPol ternary complex has a 2′,3′-dideoxy terminated 3′-end of the primer strand. Both complexes are shown in cartoon representation and the DNA is shown as sticks. The oxoG•C PrimPol ternary complex is shown in colors (molecule A of the asymmetric unit): the N-helix in dark blue, modules ModN in yellow and ModC in cyan, the DNA in gray, and the Ca^{2+} ion is shown as a light blue sphere. The oxoG residue is shown in orange and its partner C base in red. The entire unmodified T-dATP complex (PDB ID: 5L2X)[2] is colored in beige. The unmodified complex has a C4 template base and G14 primer base at the positions corresponding to the oxoG residue and its partner C14 primer base in the modified complex, respectfully. The unstructured regions in the ModN and ModC modules are shown as dashed lines. The complexes are superimposed by the ModN and ModC of PrimPol protein.

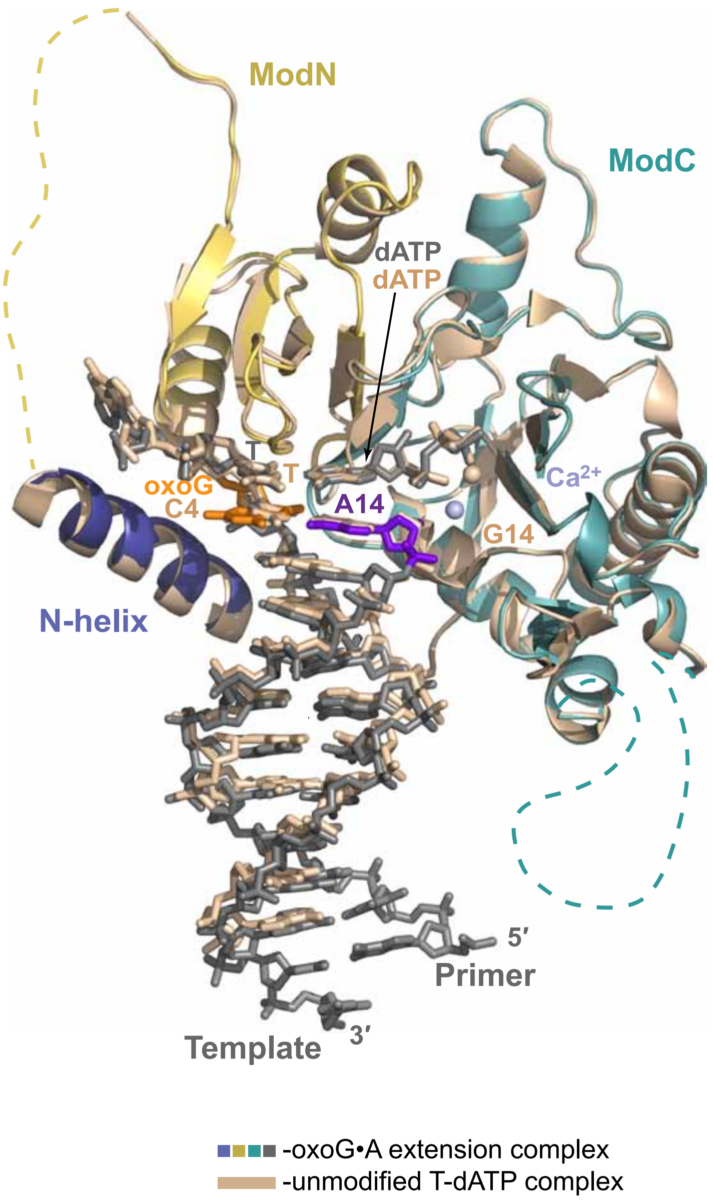

Supplementary Figure 6

Supplementary Figure 6. Comparison of the oxoG•A extension and the unmodified PrimPol ternary complexes. The oxoG•A extension complex has the 3′-deoxy terminated primer A base and the unmodified T-dATP PrimPol ternary complex has a 2′,3′-dideoxy terminated primer strand. Molecules A (with ordered N-helix) of the asymmetric unit are shown for both complexes. PrimPol protein is shown in cartoon representation and the DNA is shown as sticks. The oxoG•A PrimPol ternary complex is shown in colors: the N-helix in dark blue, modules ModN in yellow and ModC in cyan, the DNA in gray, and the two Ca^{2+} ions is shown as a light blue sphere. The oxoG residue is shown in orange and its partner C base in red. The entire unmodified T-dATP complex (PDB ID: 5L2X)[2] is colored in beige. The unmodified complex has only one Ca^{2+} 'metal B' ion, the extension oxoG•A complex carries both ions, catalytic 'A' and dNTP tail-chelated 'B'. The unmodified complex has a C4 template base and G14 primer base at the positions corresponding to the oxoG residue and its partner A14 primer base in the modified complex, respectfully. The unstructured regions in the ModN and ModC modules are shown as dashed lines. The complexes are superimposed by the ModN and ModC of PrimPol protein.

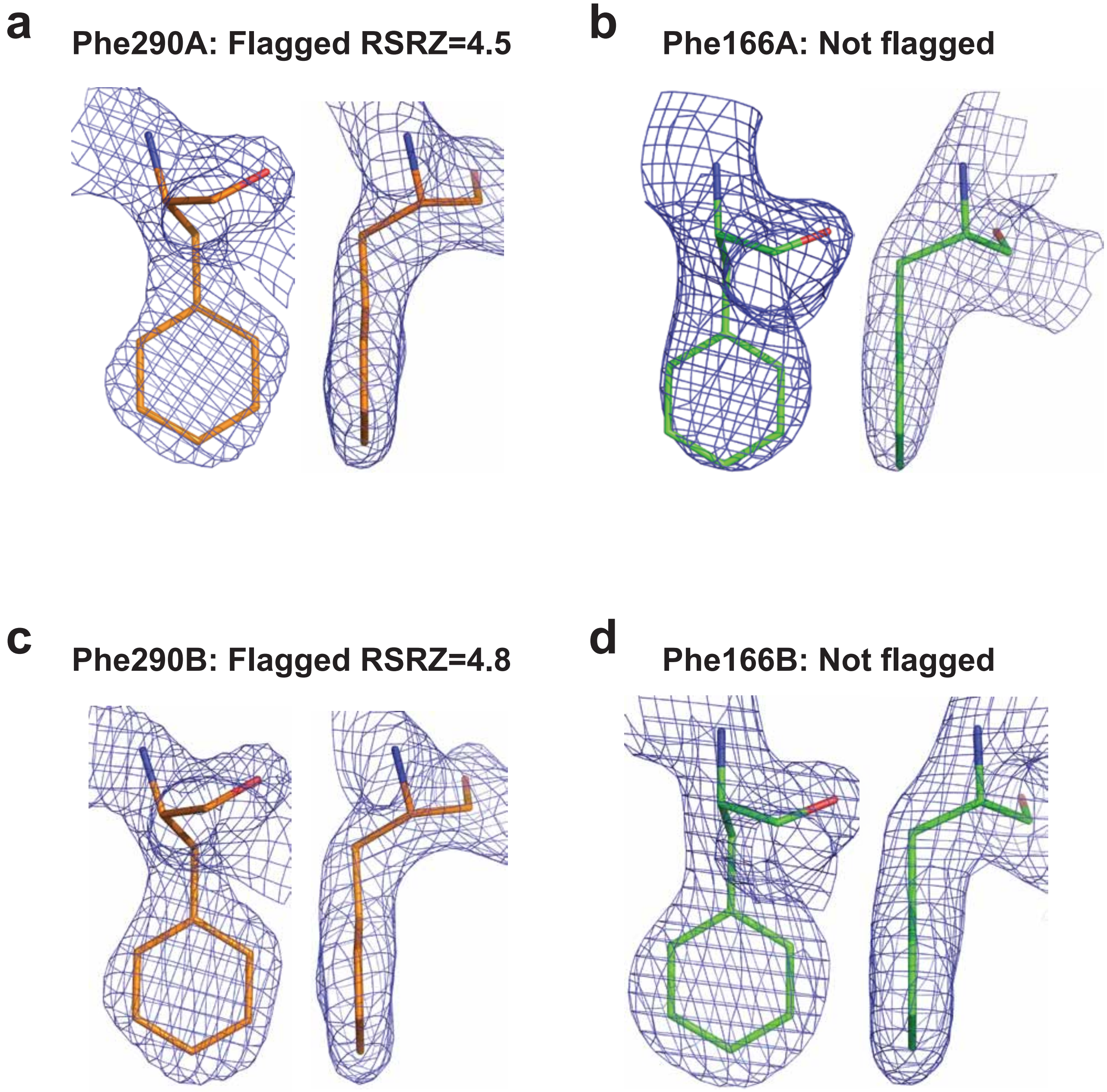

Supplementary Figure 7

Supplementary Figure 7. Electron density maps for the flagged RSRZ outlier residues Phe290 in chains A and B and comparison with the not flagged Phe166A and Phe166B in the oxoG•C extension complex. a 2Fo − Fc map (contoured at 1.0σ-level at 2.1 Å resolution and colored in blue) showing the clear electron density for the Phe290 chain A residue. The validation report, calculated upon the data deposition to Protein Data Bank (PDB), flags Phe290A as an outlier with a RSRZ=4.5. The PDB validation report states that flagged RSRZ outlier residues are those "that have a poor fit to the density." However, the 2Fo − Fc map for Phe290A is of high quality. There are no Fo − Fc difference map peaks at or near Phe290A even if countered at very low 2.0σ-level. Phenix comprehensive validation indicates a real space correlation coefficient (CC) of 0.98 for Phe290A. **b** 2Fo − Fc map for not flagged Phe166 chain A residue. **c** 2Fo − Fc map for the Phe290 chain B residue flagged as an outlier with RSRZ=4.8. Phenix comprehensive validation indicates a CC of 0.99 for Phe290B. d 2Fo − Fc map for not flagged Phe166 chain B residue.

Supplementary Table 1. DNA templates and primers used for crystallization of the oxoG-modified PrimPol complexes.

Complex name /PDB ID	Complex type (relative to the position of the oxoG lesion)	DNA template	DNA primer
oxoG•dCTP 7JK1	Insertion	5′–CA(oxoG)CGCTACCACACCCC–3′	5′–GGTGTGGTAGCG–3′
oxoG•dATP 7JKL	Insertion	5′–CA(oxoG)CGCTACCACACCCC–3′	5′–GGGTGTGGTAGCG–3′
oxoG•dATP 7JKP	Insertion	5′–CA(oxoG)CGCTACCACACCCC–3′	5′–GGGTGTGGTAGCG$_{dd}$–3′ (G$_{dd}$ is a 2′,3′-dideoxy-G)
oxoG•C 7JL8	Extension	5′–CAT(oxoG)CCTACCACACCCC–3′	5′–GGGTGTGGTAGGC–3′
oxoG•A 7JLG	Extension	5′–CAT(oxoG)CCTACCACACCCC–3′	5′–GGGTGTGGTAGGA–3′

SUPPLEMENTARY REFERENCES:

1 Frank-Kamenetskii, M. D. & Mirkin, S. M. Triplex DNA structures. *Annu Rev Biochem* **64**, 65-95, doi:10.1146/annurev.bi.64.070195.000433 (1995).

2 Rechkoblit, O. *et al.* Structure and mechanism of human PrimPol, a DNA polymerase with primase activity. *Sci Adv* **2**, e1601317, doi:10.1126/sciadv.1601317 (2016).
